# Precision education – a call to action to transform medical education

**DOI:** 10.1186/s12245-025-00819-1

**Published:** 2025-02-10

**Authors:** Wendy C. Coates

**Affiliations:** 1 University of California, Los Angeles, USA; 2 David Geffen School of Medicine at UCLA, Los Angeles, CA USA

**Keywords:** Artificial intelligence, Precision education, Competency based medical education, Master adaptive learner, Medical education, Health professions education

## Abstract

**Background:**

Institutions, departments, and individuals are increasingly facing challenges to determine how to enable their learners to acquire and curate rapidly changing knowledge and to foster the creation of lifelong learners in this information-rich digital era.

**Methods:**

Much like the Precision Medicine initiative of 2015, in which diagnostic, treatment, and preventive care target individual patients based on their genetic and environmental profiles, educators can use the same principles to create a model of “Precision Education.”

**Results:**

In this model, future facing individualizable educational infrastructure can consider innate qualities, learning style, behavior, environment, prior experience, expertise, and assessments.

**Conclusion:**

Educators can utilize Artificial Intelligence, the Master Adaptive Learner model, and key components of Competency Based Medical Education to transform the evolution of Health Professions Education to meet the individual and systemic needs of tomorrow’s learners, educators, and institutions to improve educational and clinical outcomes.

## Background

Institutions, departments, and individuals are increasingly facing challenges to determine how to enable their learners to acquire and curate rapidly changing knowledge and to foster the creation of lifelong learners in this information-rich digital era. In the early 20th century, Flexner redefined the world of medical education to include a knowledge acquisition (pre-clinical) and an application (clinical) phase [1]. In this model, the physician was expected to be a repository of knowledge who accessed and applied it appropriately in clinical situations. Learners acquired information via a combination of large group didactic lectures, small group learning experiences (e.g., anatomy lab with partners who shared a preserved human cadaver), and by relying on print textbooks and articles in journals that were available in institutional libraries or via inter-library loan. As the 21st century began, the rate of doubling of medical knowledge increased at an alarming pace, from 50 years in 1950 to about 73 days in 2021 [2, 3]. It is no longer possible for a human brain to retain nor replace information at this rate, transforming today’s clinician from a repository into a curator of knowledge. If our medical education system is to train future physicians to lead and innovate in this transformational era, adopting new strategies is imperative. These include changes in the roles of educators, learners, and the educational system [4]. For over a decade, medical educators have considered that learners process material in different ways and at different rates, making the concept of a personalized educational strategy an attractive, yet cumbersome one [5, 6]. However, with the advancement of technology and Artificial Intelligence (AI), it is likely that educators can now realize this vision. The objective of this perspective piece is to consider how medical educators may be able to utilize self-directed learning strategies, technology, and AI to develop an effective multi-modal medical education model for the rest of the 21st century.

### Drawing a parallel to precision medicine

In 2015, President Obama launched the Precision Medicine Initiative to refute the standard “one-size-fits-all” healthcare structure in favor of targeted diagnosis, prediction, treatment, and prevention based on individual patients’ genes, lifestyle, and environment using the support of AI [7–9]. Extrapolating from this concept of individualized care, educators recognized that learners are heterogeneous and that the same AI principles could define a new field, Precision Education, which first was proposed as a strategy to classify and treat children with learning disabilities then broadened to provide a new framework for education for all learners [10]. Table 1 draws parallels between Precision Medicine and Education. Table 2 is a glossary of technologic terms with examples.

**Table 1:** Parallels between precision medicine and precision education

Medicine		Education
H&P, diagnostic tests, gene mapping, lifestyle analysis, environment	Diagnosis	IQ, test scores, GPA, prior education, dashboard data, classroom behavioral observation
Disease risk factors based on aggregate data, machine learning algorithms	Prediction	Online learning logs, observed academic performance, machine learning algorithms
Match genes, environment, pathology to target treatment	Treatment	Target learning experiences to fill gaps or advance strengths
Based on genetic, environmental and lifestyle data of population	Prevention	Longitudinal assessments (human and AI) of student to guide future interventions early on

H&P, History & Physical; IQ, Intelligence quotient; GPA, Grade point average; AI, Artificial intelligence

**Table 2:** Glossary of technology terms for the medical educator [21, 23, 24]

Term (Abbreviation)	Definition	Example
Algorithm	Step-by-step set of instructions used to solve a problem	Step-by-step description of how to insert a central venous access catheter
Artificial Intelligence (AI)	Process that enables a computer to interact with the human world using perceptions that are generally associated with human traits (e.g., speech, visual interpretation, behavioral actions)	Chatbot on a website’s customer service page
Artificial Intelligence Education (AIEd)	Application of AI to the educational setting to create a unique and adaptive learning structure for each learner	Language learning app that tailors future lessons to individual learners to drill missed items and skip mastered skills
Augmented Reality (AR)	Addition of computer-based features to a real-world system	Yellow line indicating a “first-down” superimposed on a football field during a telecast
Big Data	Vast and ever-growing collection of data in a given domain	Patient databases; Aggregated standardized test scores
Decision Theory	Mathematical analysis of options to guide the best course of action that considers relative risks and benefits	Weigh the effect of an extra 20 h of studying to improve a score by 1-point on a criterion-based exam
Deep Learning	Sophisticated subset of machine learning that uses neural networks to help the computer train itself to improve its skill from its own experience and without additional human input.	Online translation platform that adjusts for grammar, syntax, and idioms instead of a literal translation from the original language
Machine Learning (ML)	Type of AI that allows computers to learn from their own experience and available data to improve their ability to perform tasks or to improve their accuracy of prediction of outcomes	Virtual assistant who can comprehend and predict needs of its human user
Natural Language Processing (NLP)	Type of AI that recognizes language-based input to process understanding	Automatic real-time captioning of the spoken word to create a written record of what was said
Neural Network	Series of interconnected datasets that interact with one another in the process of deep (machine) learning to weigh alternatives and learn from past experiences and interactions. The terminology is based on the interactive function of the human brain.	Self-driving car
Virtual Reality (VR)	A computer-generated immersive environment that may include multisensory input and enables the user to control interactivity. It is generally accessed through a technological interface	Flight simulator

### Is precision education really a new concept?

When one considers educational strategies in K-12 educational settings [11], ability-based small-group learning, such as advanced reading or mathematics students who are grouped together for fast-paced challenging work, are common. Similarly, students needing remedial attention or require dedicated support in other areas (e.g., social or executive functioning skills) are pulled from the general classroom to focus on their individual needs. In sports, there are team levels to suit any ability level (e.g., intramural, junior varsity, varsity). When students reach college, differentiated opportunities diminish, but some still exist (e.g., honors program, living/learning programs, sports). Some medical school programs fostering individualized learning exist, but often involve rigid tracks (e.g., Medical Scientist Training Program in which graduates earn both an MD and PhD, and other dual-degree programs, such as MBA, MPH, etc.). Opportunities during graduate medical education (GME) are sparse. One example is the presence of scholarly tracks targeting residents’ future career aspirations and allow for exploration and specialization during training (e.g., research, administration, education) [12]. 

### Characteristics of precision education

Current training structures are generally time-based, with defined starting and ending dates as the bookends of training. Many assume that if learners are present for the proscribed time period, they can meet the stated learning objectives, and at the completion of the time attached to the program, their training is officially complete. Some examples in the United States include four years each for high school, university, undergraduate medical education (medical school), and a specific number of years for post-graduate (residency) training that varies by discipline. In many cases, there are safeguards in place to provide extra time for learners who have not met the minimum requirements for advancement. It is likely that individuals enter their training programs with varying levels of pre-existing mastery and that they may differ in their abilities and time needed to master extant learning objectives.

In the proposed “precision education” structure, interventions are targeted to individual learners’ needs and avoid educational “wasted” time that is inherent in time-based learning structures. For example, a learner who demonstrates proficiency is not likely to benefit from learning that concept again at the novice level. This individual’s time could be more productively spent by either remediating a different skill that is lacking or by delving more deeply into the stated skill to reach an even higher level of mastery. This aligns with the concept that the medical profession involves lifelong learning. As for any educational intervention, those derived in the name of precision education must be science-based, data driven, and rigorously tested to demonstrate improved training outcomes [13–15]. When considering the adoption of AI into the learning milieu, a critical underlying concept is the reliability of the modeling AI tools. Any bias introduced during the creation phase of the proposed AI tools is likely to be compounded with the generative nature of AI, using large language models (LLM) and deep learning and deep neural networks. Thus, meticulous attention to minimizing (or eliminating) bias in tools that may have an eventual effect on patient outcomes is critical. It is prudent to ensure a system of checks and balances, in which humans periodically verify the accuracy and intervene to eliminate any bias that is discovered.

### Creating a precision education plan

An organized process to creating appropriate experiences focuses on the needs of the learners, faculty, and institution. One must first analyze learners to determine their individual needs. This may begin with an inventory of objective data (e.g., IQ tests, standardized test scores, grade point average) and continue with in situ observation of learning behaviors and performance. In many settings, the admissions/selection process may provide a foundation for this information. Some settings that have interactive learning dashboards may be able to use AI to add to the baseline learning profile. Involving the learner in this initial assessment is key and may include their self-assessment of knowledge, aspirations for future accomplishments and learning style preferences. Each learner has unique background experiences, including personal factors, prior educational experiences, and their environment. Adopting a flexible perspective enables individualized solutions that can eliminate or modify requirements for learning objectives for learners with demonstrated competency. The timing, amount of repetition of educational materials needed for mastery, the modality of learning, and the amount of faculty involvement may vary for different learners. AI monitoring strategies may facilitate and optimize these individual programs. Oversight and consistent monitoring are essential components and may include ongoing formative assessments that may require real-time adjustment of learning strategies and materials. Both immediate and intermittent formative assessments should be built into the structure and there should be transparency of expectations and measurable outcomes. In addition to human feedback, programs may choose to integrate data dashboards or electronic health record (EHR) systems to monitor progress and identify gaps [16]. Smooth implementation and maintenance of this structure requires extensive collaboration among multidisciplinary teams, learners, faculty, and information technology (IT) experts [17, 18]. Particular attention to ethical considerations to protect the learner’s privacy is critical at all stages of the learning plan, especially when data collection and use are involved.

### The master adaptive learner

The American Medical Association’s Accelerating Change Initiative described a metacognitive model, the Master Adaptive Learner (MAL), that is centered on a learner’s ability to self-regulate knowledge acquisition, decision making, and adaptive expertise to devise innovative approaches to problem solving [13]. In this model, a curious and self-motivated learner with an open mindset to growth is key. Much of the learning is self-directed and may involve seeking out appropriate sources of needed information [14]. With the plethora of available resources that are widely available, incorporation of AI in the selection of appropriate learning materials can save time and home in on resources whose learning objectives align with learner needs. The optimal master adaptive learner should demonstrate resilience and be able to excel in the face of learning challenges with appropriate support of coaches. When medical education programs or institutions are considering the incorporation of Precision Education into their outcomes strategies, a reasonable goal could be to facilitate the development of trainees who are Master Adaptive Learners. Providing exposure to the MAL model and the responsible inclusion of AI during the training phase is likely to foster this learning strategy to extend to lifelong learning activities and requirements for maintenance of certification. In addition, widespread facility with technologic learning structures is likely to increase the cohort of practitioners at an institution who are adept and can keep abreast of new developments.

### Competency based medical education

Competency based medical education (CBME) is closely aligned to the MAL model in that it is based on learner-centric attainment of pre-defined learning outcomes [4, 19]. Throughout history, it has been implemented successfully, especially in times of great national need [20], and remains a valuable opportunity for educational programs and institutions to revamp existing curricular structures [21]. By applying the concepts of MAL in conjunction with CBME during curricular reform, the infrastructure changes (e.g., cost, data management, service vs. learning needs) required can be addressed together [22]. 

Precision education and CBME are topics that span many domains, and the definition may vary based on the application. For example, in agriculture, using technology and AI for optimal crop yield and quality requires mastery of specific (and often, new) knowledge, skills, and abilities (KSA) in the workforce to operate machinery safely and predict supply chain needs [23]. Industry standards and practices in engineering rely on learners with a precision engineering education to demonstrate higher levels of proficiency and adaptability [24]. The aviation industry, once a pioneer in simulation, embraces the concept of CBME and precision education to make aviation safer [25]. 

### The call to action

Some decision makers who are in powerful positions to enact change in their administrative leadership roles that could transform their educational institutions to adopt AI for education (AIEd) may not be as facile with technological advances as their digital native counterparts and may, in fact, be acting as barriers to progress. To counteract this mismatch of power and desired reform, one of the most important actions is to create faculty development programs to educate leaders to be effective stewards of change [26]. Enlightened individuals can become “early adopters” of new technology within the limits of their own technical prowess. Institutional leaders can create a long-term vision for precision education and map out a stepwise strategy to attain their goals. Information technology (IT) experts who are invested in the program’s success and are regularly available to troubleshoot and grow the platform are key members of the team (i.e., in-house IT rather than disconnected consultants). They may be non-medical computer scientists and/or adept physicians with IT expertise [14]. A savvy educator with interest in AIEd and IT experience could pilot test a small AIEd program to develop best practices for overall curricular reform that grows by an iterative process [26]. It is important to realize that not all individuals who are labelled as “digital natives” are fluent in AI or facile with technology beyond their daily activities. To be most effective, training programs aimed at the users must also exist.

It is likely that other cultural and systematic changes will have to occur to ensure success in such a transformation. This may include the cost of infrastructure upgrades, training costs for faculty and learners, and an increased number of trained faculty needed [27]. The age-old debate between time-based and competency-based education must be reconsidered [28]. Current criteria defining the ideal medical school applicant may need to be updated to select for motivated, self-directed individuals who are likely to succeed in the MAL model. Training about AI [29], technology, and effective coaching will be necessary to reduce bias against AI and to redefine cultural norms in the education space [30]. Particular attention to defining the learner as the driver of their own education is important and will set the stage for lifelong learning strategies. One must consider strategies to incorporate AI as a learning tool and apply caution to prevent AI from taking over as the driver of learning decisions. Ethical concerns must be a cornerstone and receive deliberate attention in building AI systems, defining educational standards, and creating operational processes. Whether AI-based precision education learning is the optimal strategy moving forward is not yet known, however, the prevalence of AI in academia appears to be here to stay [31]. Educators who adopt AI principles into their curricula, including precision education, can serve as pioneers in this field and help to define when AI and precision education are superior and when more traditional educational strategies may be more reliable.

## Conclusion

The information age is in full swing, and the future of graduate medical education is at a crossroads to consider whether to adopt the model of Precision Education to ensure future physicians are equipped to be effective curators of knowledge who are digitally fluent and self-regulated learners to define the practice of medicine throughout the next several decades.

## Data Availability

No datasets were generated or analysed during the current study.
